# Evaluating the Potential Benefit of a Combined Weight Loss Program in Dogs and Their Owners

**DOI:** 10.3389/fvets.2021.653920

**Published:** 2021-04-20

**Authors:** J. Rebecca Niese, Tierney Mepham, Mirjam Nielen, Evelyn M. Monninkhof, Floor M. Kroese, Denise T. D. de Ridder, Ronald J. Corbee

**Affiliations:** ^1^Department of Population Health Sciences, Faculty of Veterinary Medicine, Utrecht University, Utrecht, Netherlands; ^2^Department of Clinical Sciences, Faculty of Veterinary Medicine, Utrecht University, Utrecht, Netherlands; ^3^Julius Center for Health Sciences and Primary Care, University Medical Center Utrecht, Utrecht University, Utrecht, Netherlands; ^4^Social, Health and Organizational Psychology, Utrecht University, Utrecht, Netherlands

**Keywords:** canine, obesity, weight loss, human-animal bond, overweight

## Abstract

**Introduction:** Little has been published on the psychological bond between the owner and the pet, and how this might influence shared habits that could lead to overweight and obesity. Another factor that could improve the effectiveness of a weight loss plan, is that the owner would see the dog as a weight loss partner and therefore this could increase the motivation to follow the assigned diet and exercise guidelines.

**Objective:** The aim of this research was to evaluate the potential mutual effects of weight loss programs for both dogs and dog owners.

**Methods:** Two studies were conducted: In the human-centered trial, 60 dog owners were enrolled, who signed up to receive dietary and exercise recommendations to lose weight themselves during an 8 week period, from which 29 were randomly assigned to also get recommendations for their dog. For the dog-centered trial, we selected 13 dog owners that wanted their dog to lose weight during a 6 week period, from which 7 were randomly assigned to also get recommendations for themselves. The average weight loss over the time period was recorded. A questionnaire was used to evaluate diet and exercise habits, as well as information about the relationship between the dog and owner.

**Results:** The average human weight loss was 2.6% in the owner+dog group (*n* = 29) and 2.3% in the owner only group (*n* = 31; *p* > 0.05). Forty percent (24/60) of the dogs in the human-centered trial were overweight. The overweight dogs in the owner+dog group (*n* = 12/29) lost 3.7% of their body weight, compared to 1.2% in the overweight dogs from the owner only group (*n* = 12/31; *p* > 0.05). In the dog-centered trial, the 7 dogs in the dog+owner group lost 8.0% of their body weight, vs. 8.3% in the six dogs in the dog only group (*p* > 0.05). The owners in the dog+owner group lost 2.5% of their body weight, compared to 0.5% in the dog only group (*p* > 0.05). In both trials owners' perceived responsibility for both their own and their dogs' weight significantly increased. In addition, habit strength regarding unhealthy feeding and exercise behaviors in relation to the dogs decreased, and self-efficacy in relation to providing the dog with healthy food and exercise increased.

**Conclusion:** Active weight loss in either dog owner or dog, seemed to lead to passive weight loss in the other, especially when some tools or guidelines were provided. These findings support mutual benefits of weight loss programs for dogs and dog owners, and support future weight loss programs to be a One Health approach.

## Introduction

Overweight has a high prevalence in people and pets in the western world. In the US, the prevalence of human obesity [i.e., a Body Mass Index (BMI) of ≥30] in 2017–2018 was 42.4% (1), and 56% of the dog population [i.e., a Body Condition Score (BCS) of ≥6 out of 9] was classified as overweight (2). In the same period in the UK, the prevalence of human obesity was 29% (3), and 59% of the dog population was overweight (4). An association between overweight in dogs and their owners has been demonstrated, as dogs rely on their owners for their food, snacks, and activity (5). Treatment of obesity consists of dietary and exercise recommendations in both people and pets, and medication and/or surgical procedures in more severe cases in people. A healthy diet for people is diverse and includes plenty of fruits and vegetables, whole-grain products, less free sugar, less salt, less saturated fats, more unsaturated fats (oils, nuts), and plenty of water (6). Exercise does not necessarily include doing sports every day, as walking 10,000 steps daily and avoiding sitting down for too long also improves physical health (7). Dogs also benefit from a combination of healthy diet and exercise. Nutritional recommendations by veterinarians include avoiding snacks, providing less food, increased dietary fiber, increased dietary protein, decreased amounts of fat, and providing plenty of water (8). Exercise for at least 30–60 min a day is recommended for dogs to improve physical health (9).

Currently, overweight and obesity in humans and in their pets are being treated separately. However, we propose that the problem of overweight/obesity in dogs and owners may benefit from a One Health approach, where both groups are targeted together. There are several indications that dog owners may experience benefit when their dogs are following a weight loss program at the same time, and vice versa. Previous research has already shown that dogs positively affect the health status of their owners. For example, one study showed that dog owners had an average of 55 min more physical activity per week compared to people without dogs (10). Another trial showed that dog owners were more likely to achieve their own walking goals (11). Beyond the obvious explanation that dogs simply need to be walked and hence stimulate physical activity, researchers have proposed that positive health effects for dog owners also result from the commitment of the owner to the dog (12), as well as increased perceived social support (11). Indeed, dogs are typically considered an important source of social support (13), which could benefit overweight people, as social support is known to positively affect weight loss attempts (14). Mutual benefits can be suggested, as in a previous weight loss study in people and pets, dogs got more exercise due to their owners' active weight loss program (15).

The majority of publications on overweight and obesity in pets and owners explore the possible association between the lifestyle of the owner and the body condition of the pet, whereas little has been published on the psychological bond between the owner and the pet, and how this might influence shared habits that could lead to overweight and obesity. Another factor that could improve the effectiveness of a weight loss plan, is that the owner would see the dog as a weight loss partner, and therefore this could increase the motivation to follow the assigned diet and exercise guidelines. The aim of this study was to evaluate the potential mutual effects of a weight loss program for both dogs and owners on each other. The primary objective was to demonstrate if an active weight loss trial in either dog or dog owners would lead to passive weight loss in the other, irrespective of offering tools and guidelines.

## Materials and Methods

All participants voluntarily applied to be enrolled in the trials and could withdraw at any time. According to Dutch legislation, all participants provided written informed consent, as required for ethical approval.

An active weight loss trial in dog owners (human-centered trial) as well as an active weight loss trial in dogs (dog-centered trial) were conducted. Both were randomized clinical trials with two-arms. Participants were recruited by social media (Facebook messages, newspapers, local news, local radio, University website, and Twitter) and distribution of posters in dog- or obesity-related areas (veterinary practices, pet stores, doggy day care centers, dog training centers, animal shelters, apartment complexes, and a physiotherapy practice).

Inclusion criteria for the human-centered trial were: being an adult owner and caretaker of a dog, having a BMI above 25.0, and agreeing to participate by signing an informed consent. The dog's BCS was not considered an inclusion criterion, as healthy-weight dogs should be able to provide peer support to a similar extent as overweight dogs. The additional inclusion criterion for the dog-centered trial was: the dog had to have a BCS of 6 or higher, and for this trial the BMI of the owner was not considered an inclusion criterion.

The only exclusion criterion for both trials was being unable to walk together with the dog for roughly one hour per day. Data was collected at the Faculty of Veterinary Medicine, Utrecht University, Utrecht, The Netherlands. Participants were invited to the clinic for personal appointments between one researcher (JRN for the human-centered trial, TM for the dog-centered trial) and one participant with their dog per visit.

### Human-Centered Trial

Prior to the first visit, all participants filled out a food diary for 1 day for themselves and their dogs, in order for the clinician/researcher to be able to provide personal advice at the end of the consultation. Participants were invited to the clinic and provided written informed consent. Thereafter, baseline measurements took place. All baseline and final measurements were taken by the same clinician/researcher that was not blinded to the group allocation. The dog owner's body weight, fat percentage, and waist circumference were measured, as this is common practice in human obesity evaluation (16). The weight and fat percentage were measured (to the nearest 0.1 cm or 0.1%, respectively) using a Soehnle Body Balance Shape F4 weighing scale. The waist circumference was measured between the pelvic bone and the last rib. Dogs were evaluated by determining their BCS, and measuring their body weight and height. The BCS was determined by palpation and observation of the ribs, waist, abdominal tuck, pelvic area, and lumbar vertebrae (17). The dog's body weight was measured using a veterinary weighing scale, and its height was measured at the withers. Baseline steps per day for dogs and their owners were determined the day after the first clinical visit (when pedometers were distributed). Owners were instructed to walk their usual amount for that particular day.

After these measurements, a questionnaire was distributed to determine participants' baseline values of parameters concerning their motivation for and expectations of the program, current eating- and exercise habits of both owner and dog, cognitive abilities such as laziness and ability to stick to long-term goals, and about the relationship between the dog and owner. All questions were answered using a Likert-scale of one to seven (1 = totally disagree/never, 4 = neutral, 7 = totally agree/always) (Appendix A in Supplementary Material). Participants were alternately assigned to owner only or owner+dog group, and got information about the group allocation (owner+dog or owner only, and the difference between the two) at the end of trial, only when they were interested. The researcher was not blinded for the group allocation. The participants were unaware of group allocation, only the general objective (i.e., effects of dogs on a human weight loss program) was explained. Participants were instructed to adhere to a dietary and exercise recommendations for an 8 week period during spring. In the owner+dog group, dietary and exercise recommendations were given for the dog owners, as well as their dogs, whereas in the owner only group, the dog owners only received diet and exercise recommendations for themselves. Owners of healthy-weight dogs in the owner+dog group were asked to strictly adhere to their dog's eating habits, as if the dog was on a diet.

Diet recommendations were given according to the information leaflet based on the recommendations of the Dutch Nutrition Center [i.e., a diverse diet including plenty of fruits and vegetables, whole-grain products, less free sugar, less salt, less saturated fats, more unsaturated fats (oils, nuts), and plenty of water (6)]. This leaflet provided information on healthy eating, limiting unhealthy foods and reducing portion sizes. Additionally, the participants were given exercise instructions [i.e., 10,000 steps per day, not sitting too long (7)].

For the owner+dog group, additional diet and exercise recommendations for the dogs were given. The dietary intervention for the dogs was based on an ideal Body Condition Score (BCS) of five out of nine and an expected weight loss of 0.5–2.0% body weight per week. To calculate the ideal body weight, each BCS point above 5 was equal to 10% excess body weight. The resting energy requirement (RER) for the ideal body weight was then calculated with the following formula: RER = (body weight in kg)^∧^0.75 × 293 kJ. Once the energy density of the food had been calculated, the RER was divided by the energy density to give a number of grams to feed the dog per day. The energy density of treats were also calculated and owners were instructed to reduce the usual food by the same amount of energy to compensate for treats [adapted from (8)].

They were also instructed to walk their dogs at least 60 min per day (9). Pedometers were distributed (Yamax SW-650 for humans and Tractive Motion Pet Activity Tracker for dogs) to track their numbers of steps during trial. Every day, the participants recorded the number of steps of themselves and the number of steps of their dog in a diary. Two weeks later, participants received a phone call in which experience so far and any issues relating the pedometers were addressed. Six weeks later (8 weeks after the first appointment), the participants came in for the final appointment. The same parameters were measured by the same researcher using the same tools, and the pedometers were returned. The final questionnaire included questions about participants' abidance by the program, reflection on their expectations, experienced changes in eating- and exercise habits of both owner and dog, and several forms of peer support that may or may not have occurred (Appendix B in Supplementary Material).

The study was powered to detect a 30% difference in success between groups at 8 weeks follow-up, based on a body weight percentage loss of 5%, a power of 0.80, and 2-tailed alpha of 0.05. On the basis of these assumptions, we required 48 participants per group (96 in total per trial).

### Dog-Centered Trial

For the dog-centered trial all measurements were similar. The only difference was that in the dog only group, the dog owners just received diet and exercise recommendations for their overweight dogs, while in the dog+owner group the owners additionally received diet and exercise recommendations for themselves. The diet and exercise recommendations were the same for the human-centered trial and the dog-centered trial. The dog-centered trial lasted for 42 days instead of 56 days for the human-centered trial.

### Analysis of Questionnaires

Most questions used a Likert scale of 1–7, however, some used a scale of 1–5, therefore, after the trial the answers of the 5-point Likert scale were translated to the 7-point Likert scale, to allow for comparison. Multi-item scales for habit strength regarding unhealthy eating and exercise behaviors (toward themselves and/or their dog, depending on group allocation), self-efficacy (toward themselves and/or their dog, depending on group allocation), human-animal bound, compliance (toward themselves and/or their dog, depending on group allocation), support from their dog, other sources of support, and if the instructions were helpful, were computed after verifying the scales' reliabilities (Cronbach's alpha ≥ 0.650). For the human-centered trial, all multi-item scales were checked for correlation using a reliability test, which resulted in a Kronbach's Alpha of ≤0.572. As this is below 0.650, the multi-scale items were not correlated and were evaluated separately. Only significant results are presented. For the dog-centered trial the multi-scale items were pooled together because of the small sample size, and median scores per multi-scale item were used for analyses and presentation. Some items are presented separately.

### Data Analysis

For the human-centered trial, adjustments were performed in measured data to attribute for different duration of participation. Outcomes in these data were translated to a duration of 55 days (8 weeks minus the first day of the first clinic visit), as participation duration varied between 47 and 78 days with a mean of 57 days. The affected parameters were weight loss of humans and dogs, waist circumference loss of humans and fat percentage loss of humans. This assumed linear loss over time, so measured outcomes were divided by the amount of days participated and then multiplied by 55.

Data analysis was performed using IBM SPSS Statistics 25. Differences between groups in continuous outcomes were analyzed using a summary independent-samples *T*-test. Additionally, to assess attribution to weight loss from different support sources (friends/family, dog, and participation in research), we analyzed the data as a cohort (irrespective of group assignment) with two measurements to increase power (when no significant difference between groups was found). For these analyses we used a linear regression and chi-squared test. A *p*-value of <0.05 was set as the level of significance.

## Results

### Human-Centered Trial

**Figure 1:**
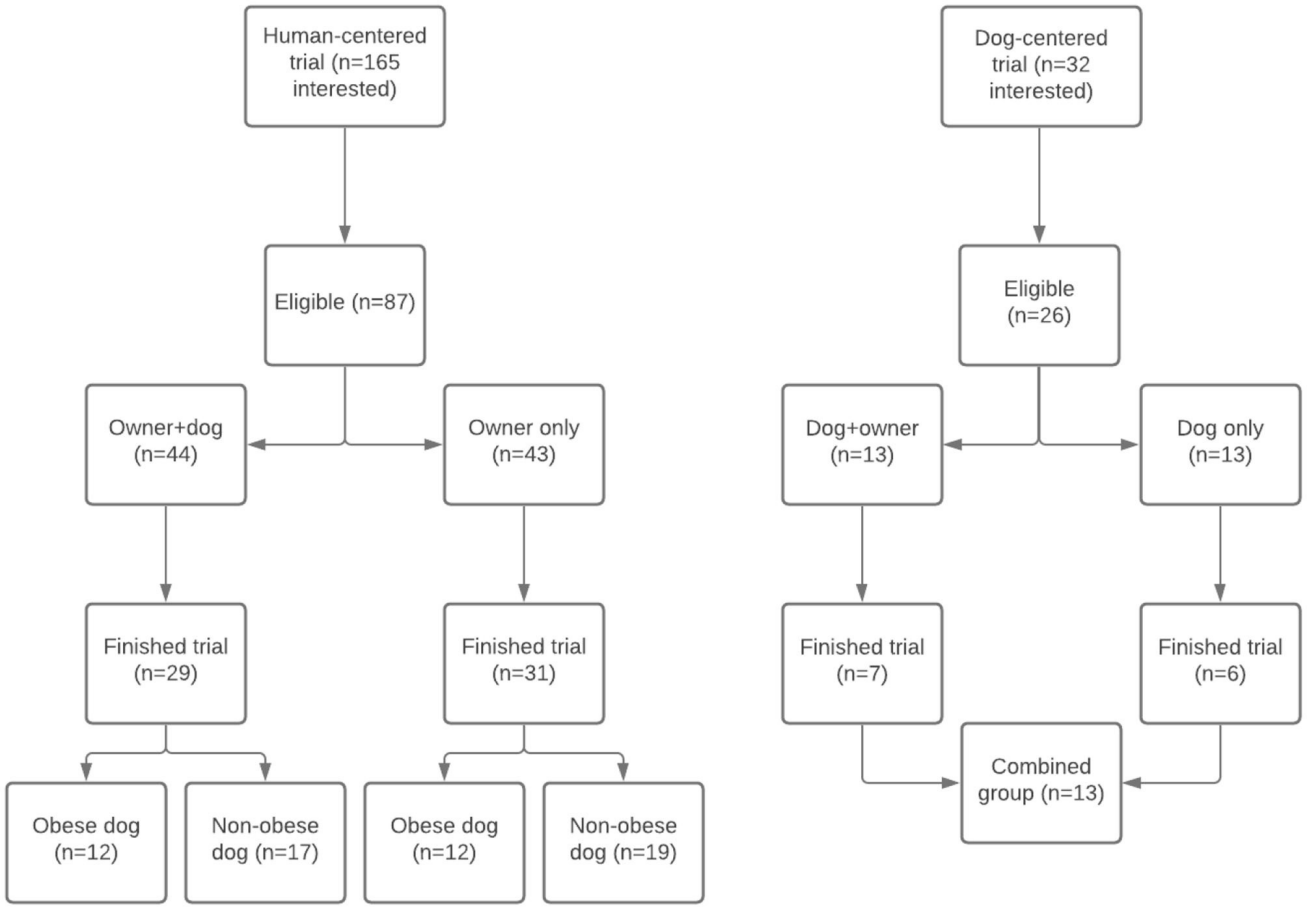
Flow chart of participants in both trials.

Of 165 interested people, 87 people appeared eligible, and were alternately allocated to the owner+dog group (*n* = 44) and owner only group (*n* = 43). Of these 87 people, 81 started the trial, and 60 finished (69%; 29 in the owner+dog group and 31 in the owner only group, respectively; Figure 1). The 21 participants that canceled their 8 weeks follow-up appointment, did so because according to them, the results were not as they expected, or they were unable to follow the given instructions. For that matter, our results need to be interpreted with caution, as we may have included mostly positive results. Only data of those who finished the intervention was included in data analysis. Comparability of both groups at intake was determined (Table 1). No large differences were found between group baseline characteristics, and these data confirmed that people in both groups needed to lose weight.

**Table 1:** Baseline characteristics human-centered trial.

	**Owner+dog group**	**Owner only group**
Number of participants	29	31
**Humans**		
Men	31%	35.5%
Women	69%	64.5%
Age	45 y/o (range 22–71)	49 y/o (range 22–74)
BMI	33.6 kg/m^2^ (SD 4.7)	31.3 kg/m^2^ (SD 3.8)
Body weight	104.8 kg (SD 16.4)	95.6 kg (SD 14.0)
Fat percentage men	35.6% (SD 6.0)	31.2% (SD 4.6)
Fat percentage women	45.3 % (SD 8.1)	41.5% (SD 6.9)
Waist circumference men	122.6 cm (SD 9.3)	116.1 cm (SD 8.0)
Waist circumference women	110.0 cm (SD 12.1)	104.4 cm (SD 9.1)
**Dogs**		
BCS dog	5.4 (SD 1.3)	5.1 (SD 1.1)
Dogs with BCS 4–5	55.2%	51.6%
**Self-reported on day 2**		
Steps per day owner	7,166 steps (SD 3,287)	7,438 steps (SD 2,355)
Steps per day dog	3,887 steps (SD 1,001)	3,375 steps (SD 1,287)

*Mean values are given with either range or standard deviation (SD). No significant differences were found between groups*.

A mean weight loss of 2.6% (SD = 2.3) was achieved in the owner+dog group, and 2.3% (SD = 2.2) in the owner only group. Weight loss within groups was statistically significant, but did not differ significantly between groups (Table 2). The number of steps for both dog owners and dogs increased significantly in both groups, but there was no significant difference between groups. Dog owners in the owner+dog group increased their number of steps from 7,170 to 9,450 steps per day vs. 7,440 to 9,770 steps per day in the owner only group, and their dogs increased their number of steps from 3,890 to 4,450 steps per day vs. 3,380 to 3,960 steps per day, respectively.

**Table 2:** Weight loss results in dog owners of the human-centered trial.

**Group**	**Start weight (SD)**	**End weight (SD)**	**Percentage lost (SD)**
Owner+dog	104.8 (16.38)	102.2 (16.81)	2.6% (2.27)
Owner only	95.6 (14.05)	93.3 (13.37)	2.3% (2.17)

*Weight loss results, in the owner+dog group and the owner only group, adjusted for 55 days of participation, mean and standard deviation (SD). Significant differences within groups, but not between groups*.

Table 3 shows the other weight loss parameters, the effects of the interventions on the dogs, and the perceived peer support in both groups. Both men and women in both groups lost a significant amount of fat and waist circumference. No differences between groups/genders were found, only within groups. Both healthy-weight and overweight/obese dogs participated in trials. To evaluate the effect of weight loss, we only included the 24 overweight/obese dogs (12 in each group; Figure 1). Dogs in the intervention group lost a mean of 3.7% (SD = 5.1) body weight in 55 days, and dogs in the control group lost 1.2% (SD = 3.5). This weight loss resulted in a lower mean BCS for each group. No statistically significant difference was found between groups, but in the intervention group, the percentage of weight loss for the dogs was statistically significant, whereas in the control group it was not.

**Table 3:** Other weight loss parameters, effects on overweight dogs, and perceived peer support, human-centered trial.

**Group**	**Fat percentage at start men (SD)**	**Fat percentage at end men (SD)**	**Percentage lost men (SD)**	**Fat percentage at start women (SD)**	**Fat percentage at end women (SD)**	**Percentage lost women (SD)**
**FAT PERCENTAGE LOSS**						
Owner+dog (9 men, 20 women)	35.6 (6.05)	32.6 (4.53)	3.0 (5.0)	45.3 (8.06)	42.7 (7.94)	2.7 (3.13)
Owner only (11 men, 20 women)	31.2 (4.63)	28.3 (4.12)	2.9 (1.92)	41.5 (6.94)	40.0 (5.84)	1.5 (3.26)
**Group**	**Waist circumference at start men (SD)**	**Waist circumference at end men (SD)**	**Percentage lost men (SD)**	**Waist circumference at start women (SD)**	**Waist circumference at end women (SD)**	**Percentage lost women (SD)**
**WAIST CIRCUMFERENCE LOSS**						
Owner+dog (9 men, 20 women)	122.6 (9.33)	114.23 (15.76)	6.7 (10.99)	110.0 (12.07)	104.9 (13.85)	4.7 (6.40)
Owner only (11 men, 20 women)	116.1 (7.98)	109.3 (8.58)	5.8 (5.98)	104.5 (9.11)	93.2 (13.91)	10.7 (10.77)
**Group**	**Weight at start (range)**	**Weight at end (range)**	**Percentage lost (SD)**	**BCS at start (SD)**	**BCS at end (SD)**	**Percentage healthy weight dogs at the end (difference)**
**EFFECTS OF THE INTERVENTIONS ON THE OVERWEIGHT DOGS**						
Owner+dog (12)	22.1 (4.9–41.2)	21.1 (4.8–38.3)	3.69 (5.063)	6.8 (0.75)	5.8 (0.84)	75.9 (+20.7)
Owner only (12)	27.3 (3.6–64.0)	26.9 (3.7–63.6)	1.17 (3.503)	6.1 (0.30)	5.6 (0.52)	71.0 (+19.4)
**Group**	**Support from friends/family (SD)**	**Support from participating in research (SD)**	**Support from dog (SD)**	**Dog supported more than/equal to friends/family (SD)**		**Dog supported more than/equal to participating in research (SD)**
**PERCEIVED PEER SUPPORT (QUESTIONNAIRE)**						
Owner+dog (29)	4.6 (1.97)	5.5 (1.76)	4.9 (1.79)	5.0 (1.69)		4.6 (1.81)
Owner only (31)	4.2 (1.33)	5.6 (1.12)	4.8 (1.33)	4.9 (1.75)		4.4 (1.45)

*Fat percentage loss, weight circumference loss and dog's weight loss have been adjusted for 55 days of participation, mean and range (Range) or standard deviation (SD). Perceived peer support was tested in the questionnaire on a scale of 1–7 (1 is totally disagree, 4 is neutral, and 7 is totally agree)*.

Questionnaire data of the whole group combined revealed a decrease in laziness from mean 3.7 (SD = 0.2) to 3.0 (SD = 0.2; *p* = 0.006). The unhealthy eating habits decreased [love for snacks from mean 5.4 (SD = 0.2) to 4.9 (SD = 0.2; *p* = 0.002); thoughtless snacking from mean 5.1 (SD = 0.2) to 4.2 (SD = 0.2; *p* < 0.001); snack frequency from mean 4.5 (SD = 0.2) to 3.2 (SD = 0.2; *p* < 0.001)], whereas healthy eating habits increased from mean 3.7 (SD = 0.2) to 4.9 (SD = 0.1; *p* < 0.001). Avoiding snack temptation increased from mean 2.6 (SD = 0.2) to 3.7 (SD = 0.2; *p* < 0.001). Healthy exercise habits increased from mean 4.6 (SD = 0.1) to 4.9 (SD = 0.1; *p* = 0.004). The final questionnaire revealed that in both groups dog owners experienced healthier food and exercise habits toward their dogs (mean 4.5 SD = mean 1.8, and 5.3 SD = 1.7, respectively).

Both groups of dog owners noted to have experienced peer support by their family/friends, dogs, and by the fact that they were participating in a research project, as all means are above neutral point 4 (Table 3). Family/friends scored lowest in both groups, followed by support from the dog, however, there was no statistically significance between these support sources. Considering no significant differences between groups for perceived peer support have been found, both groups have been combined for further analysis (Table 4). We found in linear regression analyses that only the motivation from participating in the research project significantly contributed to percentage weight loss (*p* = 0.008).

**Table 4:** Comparison of sources of support (all participants combined), human-centered trial.

	**Support friends/family**	**Support dog**	**Support research**
Mean answer	4.4 (1.55)	4.8 (1.57)	5.5 (1.46)

*The mean and standard deviation (SD) answers to the three sources of support: “dog,” “friends/family,” and “participating in research,” on a scale of 1 to 7, where 1 is “totally disagree,” 4 is “neutral,” and 7 is “totally agree.” All answers were included regardless of group allocation, as there was no significant difference between groups in any measured parameter*.

### Dog-Centered Trial

A total of 32 potential participants contacted the researcher. Of these potential candidates, 13 actually participated in the pilot, six requested more information, after which they stopped replying to emails, three agreed to participate but stopped replying before having made an appointment, four agreed to participate but changed their mind before having made an appointment due to personal circumstances, and six did not meet the inclusion criteria. Only data of those 13 candidates who finished the trials was included in exploratory analysis (Figure 1). Of the 13 owners that participated, 11 were female and 2 were male. The ages ranged from 24 to 61 years old with a median of 47.5 years. Of the 13 dogs that participated, six were female and seven were male. The median age was 5 years old, ranging between 2 and 9 years and the mean BCS was 7.7 (SD = 0.8). Four of the dogs were Labradors, five were mixed breeds, and there was one Golden Retriever, one Kooikerhondje, one Jack Russell, and one Tibetan Terrier. The measured weight loss of the dogs in percentages of initial body weight was significant within groups, but not between groups, with a mean reduction of 6.0% (SD = 4.6) in the dog+owner group and 6.2% (SD = 4.0) in the dog only group. The reduced BCSs were also not different between groups, with a mean reduction of 0.8 points (SD = 0.6) in the dog+owner group, and 0.7 points (SD = 0.5) in the dog only group. The percentage of weight loss measured in the owners had a mean of 1.9% (SD = 2.0) in the dog+owner group and 0.4% (SD = 0.6) in the dog only group, which was not significantly different.

The measured activity of the dogs did not differ significantly between the groups. The mean of the dog+owner group was 3,809 steps per day (SD = 911), whereas that of the dog only group was 3,918 steps per day (SD = 1,857). The median number of steps made by the owners in the owner+dog group was 11,348 per day (range = 1,061 to 23,027), which was more than recommended for this trial. The daily steps were not measured in the dog only group, however in earlier studies averages have been measured around 9,500 steps per day (18), much less than the dog+owner group in this study.

The perceived responsibility for the weight loss, the habit strength, and the perceived self-efficacy of the owner for them self and their dog were measured before and after participating in the pilot for both the dog+owner group and the dog only group (Table 5). The owner's perceived responsibility, increased significantly for both the weight of the owner, and for the weight of the dog. There was no significant difference between groups, so the results were pooled together to increase sample size. The perceived habit strength regarding unhealthy feeding and exercise behaviors, decreased significantly in the habits related to the dogs, however, the habits in relation to the owner's diet did not decrease significantly. The perceived self-efficacy of the owner for the weight loss of their dog, increased significantly. The difference in perceived self-efficacy for the owner's weight was almost significant (*p* = 0.056). When asked about the perceived effects of the study for the dog, owners in the dog+owner group scored themselves consistently worse than the owners in the do only group, however these differences were not significant. When asked whether they kept to the guidelines provided, the mean for the dog+owner group was 5.0 (SD = 2.4) compared to 6.2 (SD = 1.0) in the dog only group (*p* = 0.317). Although the question “This program had a beneficial effect on the health of my dog” differed only slightly in favor of the dog only group with a mean of 5.7 (SD = 2.3) compared to 5.5 (SD = 2.5) in the dog+owner group (*p* = 0.908), the question. “My dog has become healthier in the recent period” gave a mean of 6.7 (SD = 0.5) in the dog only group compared to 5.3 (SD = 2.3) in the dog+owner group (*p* = 0.188). However, when asked about the perceived effects on their own health “This program has motivated me to lose weight,” the difference was in favor of the dog+owner group with a mean of 5.6 (SD = 1.3) compared to 3.8 (SD = 1.8) in the dog only group (*p* = 0.07).

**Table 5:** The mean of perceived responsibility, strength of habit, and self-efficacy of (all participants combined), dog-centered trial.

	**Day 0 (SD)**	**Day 42 (SD)**	***p*-value**
Responsibility—dog	5.4 (1.4)	6.2 (1.2)	0.017
Responsibility—owner	6.1 (0.8)	6.9 (0.3)	0.036
Strength habit—dog	3.2 (1.8)	2.5 (1.6)	0.040
Strength habit—owner	3.1 (1.4)	2.8 (1.3)	0.398 (NS)
Self-efficacy—dog	4.3 (1.9)	5.7 (1.1)	0.022
Self-efficacy—owner	3.8 (1.8)	5.3 (1.5)	0.056 (NS)

*The mean and standard deviation (SD) of perceived responsibility, strength of habit and self-efficacy of all participants in the active weight loss trial in dogs (n = 13) at the start and end of the trial on a scale of 1 to 7, where 1 is “totally disagree,” 4 is “neutral,” and 7 is “totally agree.” All answers were included regardless of group allocation, as there was no significant difference between groups in any measured parameter. NS, not significant; dog, as perceived in relation to the pet; owner, as perceived in relation to the owner*.

## Discussion

This study aimed to explore the mutual effects of a weight loss program for both dogs and owners on each other. Based on power analysis at least 48 dog-owner pairs should have been included in each of the 4 groups. In the two conducted trials, the groups finally consisted of 29 vs. 31 pairs, and 7 vs. 6 pairs, so the trials were underpowered. The explanations can be found in unsuccessful recruitment. It was a challenge to get overweight people to a veterinary teaching hospital for treatment of their own overweight. Admitting to be overweight, and finally taking the step to do something about it is already a barrier, especially men experience barriers to start a weight loss program, which explains the lower number of men as participants (19). Surprisingly, even less people wanted their dogs to be enrolled in the weight loss trial. Unfortunately, a lot of pet owners do not recognize their dog being overweight, and if they do, they often do not consider it a condition that needs treatment (20, 21).

In this study, a mean percentage weight loss of 2.4% or 2.4 kg in 55 days (8 weeks) was found when combining both groups in the active weight loss trial in dog owners. This percentage is higher than can be expected based on previous studies evaluating the effects of diet and exercise instructions on weight loss in people. A meta-analysis determined an average of 5% (95% CI, 3.6–6.5) loss of body weight in 52 weeks (22). On the other hand, a 52 weeks is very different from an 8 weeks trial, as it also implies a higher demand for maintaining a healthy life style for a longer period of time. Another study evaluated weight loss through self-help vs. through commercial weight loss strategies found a median amount of weight loss of 2.7 kg (range = 1.5–4 kg) in a 13 weeks period (23). In the dog-centered trial, the dog owners lost on average 2.5 kg in the dog+owner group, which was similar to the amount of weight loss in the human-centered trial, whereas dog owners in the dog only group lost an average of 0.5 kg. The dogs lost on average 8.0% in the dog+owner group and 8.3% in the dog only group, respectively, in an 8 week period (i.e., 1% per week) in the dog-centered trial, and 3.7% in the owner+dog group, and 1.2% in the owner only group, respectively, in the human-centered trial (i.e., 0.3% per week). The 1% per week is a bit higher compared to levels reported in other active weight loss trials in dogs. A cohort study evaluating the success of controlled weight loss programs for obese dogs reported an average of 0.7% weight loss per week in the dogs that completed the weight loss trials (8). The 0.3% per week in the active weight loss trial in dog owners, is similar to a trial demonstrating 15% weight loss in dogs when their owners underwent a weight loss program in 52 weeks (i.e., 0.3% per week) (15).

No significant differences in weight loss parameters between the groups in both studies were found. This might be due to mutual benefits, which narrows the potential differences between groups, but it can also be due to various limiting factors. First and foremost, the group size for these trials was too small according to the power calculations to observe a significant difference between groups. It is also possible that the study's set-up did not create a big enough contrast between the intervention- and control group protocols. This was supported by the same observed effect in primary- and secondary outcomes in both groups. Furthermore, recall bias and social desirability bias most likely played a role in answering the questionnaires. A questionnaire was the only option to find out whether participants experienced peer support, but participants were aware of the research questions which may have caused socially desirable answers. Also, if dogs had a healthy weight, some questions concerning support and eating/exercise were skipped by some participants. Sometimes participants answered 1 (totally disagree/never), or sometimes 4 (neutral) to note “no difference because my dog is on healthy weight.” Even though this was adjusted for in data analysis by only taking into account the answers from owners with an overweight dog (which decreased sample size for that particular question), it may have interfered with resulting means. The translation of the Likert scores from 5-point to a 7-point scale after could have influenced the results of the questionnaires. As all measurements were performed by one researcher who was aware of group allocation and all available baseline data, it is possible that information bias have influenced the measurements. Most measurements were performed using weighing scales and, as such, are not affected. Selection may have played a role as well, as only participants who signed up for a weight loss program were observed. These people were highly motivated to work on their physique, which may have resulted in more weight loss. Also the baseline measurement of activity could have been biased, as it was only based on 1 day of activity just after the first visit to the clinic, and the participants were probably aware that they had to demonstrate an increase in activity over time. Sixty of the 87 participants finished trials (69%). In the human-centered trial, participants canceled their 8 weeks follow-up appointment mainly because results were not as they expected. This suggests we might have overestimated weight loss parameters. Lastly, the duration of the experiment may have played a role in interpreting the results. Six to eight weeks may not have been long enough to see any effects of the dog's peer support. Especially when attempting to compare to literature, 6–8 weeks follow-up is limited. On the other hand, the shorter time frame could have ensured a greater commitment on the part of the owners, which might not have happened if the program ran for 52 weeks.

No peer support from friends/family was found to affect the results of this trial, even though literature is adamant that peer support from family/friends affects weight loss. This is possibly due to the different types of peer support that can be distinguished. Literature shows controversy in dyadic support (14), but also demonstrated that group support can result in twice as much weight loss (24, 25). This study used dyadic support, and did not find a relationship between support from friends/family or dogs and weight loss results. However, support from dogs and friends/family did not differ significantly. Therefore, future studies should have a longer follow up period to allow for better comparison with literature.

Dogs appear to benefit from their weight losing owner, as the amount of dogs with a healthy BCS increased from 53.3 to 73.3% (both groups in the human-centered trial combined). This difference was statistically significant, also in the owner only group that did not receive weight loss instructions for their dog. The same is true for the benefit of the weight loss program for the dogs, as their owners also lost weight, especially when they also got some tools or guidelines (0.4% of their body weight and 1.9% of their body weight, respectively). Despite the fact that most participants mentioned to perceive peer support from dogs as well as from family/friends, this support did not significantly impact weight loss. The participants mentioned to perceive more support from their dogs compared to family/friends, however, the only significant motivator was the fact that they were participating in a research project. This is probably caused by the fact that both participants and researchers will be faced with the results (fear of embarrassment), because some participants wanted to achieve more weight loss than the others (competiveness), and because participants took participating in research very seriously (responsibility). This is confirmed by the high percentage of drop-outs (31%), amongst whom the most common reason for not returning to clinics to finish trials was the fact that weight loss had not occurred as expected. However, the support from participating in a research project does not explain any potential difference between this study's result and those found in literature, as this same support was present in all other weight loss studies. Perhaps the pedometers, which were mentioned as a form of support by some, were not included in other studies and may have contributed to the results in this study. The number of steps increased during the trial in all groups, and it appeared that setting a goal for more steps and measuring this with pedometers was effective. Furthermore, dog owners preferred to increase the number of steps (at least partly) together with their dogs. Another option is that participants benefited from peer support by the dog (and friends/family), but it was simply not found in this study due to various limitations, which is supported by the fact that we found no support from friends/family although it is known that other studies found this type of support. Considering we found friends/family and dogs supported similarly, it would be interesting to continue research in this field to confirm that dogs can also provide peer support.

The findings from the evaluation questionnaire hint toward a perceived effect of peer support by the dog on the overall health of the owner. This effect was expected due to the increased perceived social support of the dog (the weight loss partner) (13, 26) and the increased physical activity (12, 27). The perceived responsibility increased for both the owner and the dog during the trials, which could provide increased motivation for a long term weight loss plan. The perceived habit strength regarding unhealthy feeding and exercise behaviors in relation to both dogs and themselves decreased during the trials. The habit strength for giving treats and table scraps is a major underlying cause of overweight and obesity in dogs (28, 29), so this change in habit strength could contribute to the shift to a healthy lifestyle for the pet. There was also a significant increase in self-efficacy for the dog and a tendency to increased self-efficacy for the owner, which can improve the overall weight loss in overweight owners (30).

Although peer support from the dog did not significantly contribute to weight loss using current data (nor did peer support from friends/family), dogs and owners significantly lost weight during the 6 or 8 week intervention period. Support from participating in research contributed significantly to weight loss, and dogs seem to benefit from their weight-losing owner and vice versa. This is demonstrated by weight loss in dogs and dog owners in all of the groups, and by the change in habit strength and perceived self-efficacy. Considering the prevalence of obesity in humans and dogs alike, these study results show a promising option to tackling both issues at the same time. Further research with a larger group and longer intervention duration/follow-up is required for a more accurate outcome and comparison to weight-loss programmes in literature.

## Conclusion

Active weight loss in either dog owner or dog, seemed to lead to passive weight loss in the other, especially when some tools or guidelines were provided. These findings support mutual benefits of weight loss programs for dogs and dog owners, and support future weight loss programs to be a One Health approach.

## Data Availability Statement

The raw data supporting the conclusions of this article will be made available by the authors, without undue reservation.

## Ethics Statement

Ethical review and approval was not required for the study on human participants in accordance with the local legislation and institutional requirements. The patients/participants provided their written informed consent to participate in this study. Ethical review and approval was not required for the animal study because this study included dogs that had to lose weight or had to maintain weight. If weight loss was needed, treatment was similar to admitted patients in the hospital, and closely monitored. Therefore, no ethical approval was needed under local legislation. Written informed consent was obtained from the owners for the participation of their animals in this study.

## Author Contributions

JN conducted the human-centered trial, TM conducted the dog-centered trial. MN, EM, FK, and DR were involved in study design and data analysis. RC was the supervisor of the trials and also involved in study design and data analysis. All authors were involved in drafting and adjusting the manuscript.

## Conflict of Interest

The authors declare that the research was conducted in the absence of any commercial or financial relationships that could be construed as a potential conflict of interest.

## Supplementary Material

Appendices A, B are the questionnaires that were used, translated in English.
