# Supplementary material for: Evaluating the Potential Benefit of a Combined Weight Loss Program in Dogs and Their Owners
Source: Front Vet Sci. 2021 Apr 20;8:653920. doi: 10.3389/fvets.2021.653920 (PMC8093810; doi:10.3389/fvets.2021.653920)
Supplement: Supplementary file 1 [file Data_Sheet_1.docx]

Appendix A Questionnaires at baseline

Human-centered trial visit 1

**Questions about the owner themselves**

| Quote | Totally disagree |  |  | Neutral |  |  | Totally agree |
| --- | --- | --- | --- | --- | --- | --- | --- |
| I know a lot about healthy diets |  |  |  |  |  |  |  |
| I know a lot about healthy exercise |  |  |  |  |  |  |  |
| I can resist temptations |  |  |  |  |  |  |  |
| My eating habits are healthy |  |  |  |  |  |  |  |
| I am lazy |  |  |  |  |  |  |  |
| I’m good at achieving long term goals |  |  |  |  |  |  |  |
| I like to snack |  |  |  |  |  |  |  |
| I sometimes don’t realize I’m snacking |  |  |  |  |  |  |  |
| I will stick to the protocol |  |  |  |  |  |  |  |

How often do you snack?

- More than 3 times per day
- Once to 3 times per day
- Once a day
- Irregularly
- Never

Do you walk/cycle/do sports (alone or with your dog)?

- Yes, daily for more than 3 hours
- Yes, daily for 1 to 3 hours
- Yes, daily for 1 hour
- Hardly
- Never

**Questions about the dog**

| Quote | Totally disagree |  |  | Neutral |  |  | Totally agree |
| --- | --- | --- | --- | --- | --- | --- | --- |
| My dog is healthy |  |  |  |  |  |  |  |
| My dog gets treats |  |  |  |  |  |  |  |

**Questions about the human-animal bound**

| Quote | Totally disagree |  |  |  |  |  | Totally agree |
| --- | --- | --- | --- | --- | --- | --- | --- |
| My dog is my best friend |  |  |  |  |  |  |  |
| I talk to my dog |  |  |  |  |  |  |  |
| I think I will lose weight with my dogs |  |  |  |  |  |  |  |
| My dog supports me when I struggle |  |  |  |  |  |  |  |
| My dog experiences support from me |  |  |  |  |  |  |  |
| I am responsible for my dog’s weight |  |  |  |  |  |  |  |
| My dog snacks when I do |  |  |  |  |  |  |  |
| Sometimes I don’t realize I give my dog a treat |  |  |  |  |  |  |  |
| My dog understands me |  |  |  |  |  |  |  |
| Having a close watch on my dog’s weight also helps me to watch my own weight |  |  |  |  |  |  |  |

What do you feed your dog? (In percentage per day)

______ Dry food

______ Raw food

______ Sausages

______ Canned food

______ Table scraps

______ Home cooked

______ Other, please specify:

How often does your dog get a treat?

- More than 3 times per day
- Once per day
- Once per week
- Irregular
- Never

How do you feed your dog?

- Once a day, a fixed amount
- Twice a day, a fixed amount
- Three or more times a day, a fixed amount
- Ad libitum

Does the dog get leash walks?

- Yes, daily less than 1 hour
- Yes, daily 1 to 3 hours
- Yes, daily more than 3 hours
- No, the dog walks on the premises (in the garden/surroundings)
- No

Dog-centered trial visit 1

**Questions about the owner themselves**

What is your body weight (in kilograms)?

How tall are you (in centimeters)?

**Questions about the dog**

Sex

- Male
- Female

Neutered

- Yes
- No

Age in years:

Breed:

Body weight in kilograms:

Body condition score:


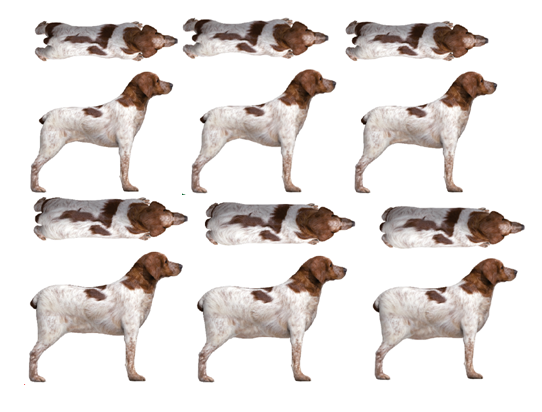


| **Questions about human-animal bond** | Totally disagree | Disagree | Neutral | Agree | Strongly agree |
| --- | --- | --- | --- | --- | --- |
| My dog is more important to me than any person in the world |  |  |  |  |  |
| I share my feelings and concerns with my dog |  |  |  |  |  |
| Dogs should have the same rights as other family members |  |  |  |  |  |
| My dog is my best friend |  |  |  |  |  |
| My feelings toward people are influenced by their attitude towards my dog |  |  |  |  |  |
| I love my dog, because (s)he is more loyal to me than any person in the world |  |  |  |  |  |
| I love to share pictures of my dog |  |  |  |  |  |
| My dog is more than just a pet |  |  |  |  |  |
| I love my dog because (s)he never judges me |  |  |  |  |  |
| My dog knows when I’m feeling down |  |  |  |  |  |
| I talk a lot with other people about my dog |  |  |  |  |  |
| My dog understands me |  |  |  |  |  |
| Loving my dog keeps me healthy |  |  |  |  |  |
| Dogs earn the same amount of respect as people |  |  |  |  |  |
| Me and my dog are very close |  |  |  |  |  |
| I’ll do almost anything to care for my dog |  |  |  |  |  |
| I often play with my dog |  |  |  |  |  |
| My dog is my best companion |  |  |  |  |  |
| My dog makes me feel happy |  |  |  |  |  |
| My dog is part of my family |  |  |  |  |  |
| I am very much attached to my dog |  |  |  |  |  |
| Owning a dog contributes to my wellbeing |  |  |  |  |  |
| I consider my dog as a friend |  |  |  |  |  |

|  | Totally disagree |  |  | Neutral |  |  | Totally agree |
| --- | --- | --- | --- | --- | --- | --- | --- |
| My dog sleeps in my bed |  |  |  |  |  |  |  |
| I like to talk to my dog |  |  |  |  |  |  |  |
| I have conversations with my dog |  |  |  |  |  |  |  |
| I spend a lot of time preparing my dog’s meal |  |  |  |  |  |  |  |
| I am willing to spend a lot of money to improve my dog’s looks |  |  |  |  |  |  |  |

I see my dog as a:

- Animal
- Friend
- Family member
- Child
- Baby
- Other, please specify: ____________________

| Responsibility | Totally disagree |  |  | Neutral |  |  | Totally agree |
| --- | --- | --- | --- | --- | --- | --- | --- |
| I am disappointed if I am unable to let my dog lose weight |  |  |  |  |  |  |  |
| It is important for me that my dog loses weight |  |  |  |  |  |  |  |
| It bothers me if I will not succeed in letting my dog lose weight |  |  |  |  |  |  |  |

| Responsibility | Not at all |  |  | Neutral |  |  | Completely |
| --- | --- | --- | --- | --- | --- | --- | --- |
| To which extend do you feel responsible for your dog’s weight? |  |  |  |  |  |  |  |
| To which extend does your behavior drives your dog’s weight? |  |  |  |  |  |  |  |
| To which extend do you feel responsible for your own weight? |  |  |  |  |  |  |  |
| To which extend does your behavior drives your own weight? |  |  |  |  |  |  |  |

| Self-efficacy towards themselves | Totally disagree |  | | Neutral | |  | | Totally agree |
| --- | --- | --- | --- | --- | --- | --- | --- | --- |
| I can resist temptations |  |  | |  | |  | |  |
| I find it difficult to stop bad habits |  |  | |  | |  | |  |
| I am lazy |  |  | |  | |  | |  |
| I say inappropriate things |  |  | |  | |  | |  |
| I sometimes do things that are bad to me for pleasure |  |  | |  | |  | |  |
| I refuse to do things that are bad to me |  |  | |  | |  | |  |
| I wish I had more discipline |  |  |  | |  | |  | |
| People say I am very disciplined |  |  |  | |  | |  | |
| Pleasures distract me to finish my (home)work |  |  |  | |  | |  | |
| I find it difficult to focus |  |  |  | |  | |  | |
| I am able to achieve long term goals |  |  |  | |  | |  | |
| Sometimes I cannot stop myself from doing something which is bad to me |  |  |  | |  | |  | |
| I often do things without thinking about possible alternatives |  |  |  | |  | |  | |

| Habit-strength towards dog | Totally disagree |  |  | Neutral |  |  | Totally agree |
| --- | --- | --- | --- | --- | --- | --- | --- |
| I often give my dog treats |  |  |  |  |  |  |  |
| I give my dogs treats without being aware of it |  |  |  |  |  |  |  |
| I give my dog treats without thinking |  |  |  |  |  |  |  |
| I give my dog treats without needing a reminder |  |  |  |  |  |  |  |
| It feels strange if I don’t give my dog treats |  |  |  |  |  |  |  |
| It will take effort not to give my dog treats |  |  |  |  |  |  |  |
| Giving my dog treats is part of my daily/weekly routine |  |  |  |  |  |  |  |
| Giving my dog treats is typically me |  |  |  |  |  |  |  |
| Giving my dog treats is something I have always done |  |  |  |  |  |  |  |

| Habit-strength towards themselves | Totally disagree |  |  | Neutral |  |  | Totally agree |
| --- | --- | --- | --- | --- | --- | --- | --- |
| Snacking is something I often do |  |  |  |  |  |  |  |
| Snacking is something I automatically do |  |  |  |  |  |  |  |
| Snacking is something I do without being aware of it |  |  |  |  |  |  |  |
| I feel awkward if I don’t take a snack |  |  |  |  |  |  |  |
| Snacking is something I do without thinking |  |  |  |  |  |  |  |
| To stop snacking will be an effort for me |  |  |  |  |  |  |  |
| Snacking belongs to my daily/weekly routine |  |  |  |  |  |  |  |
| I start snacking before thinking about it |  |  |  |  |  |  |  |
| To stop snacking will be difficult for me |  |  |  |  |  |  |  |
| I start snacking before being aware of it |  |  |  |  |  |  |  |
| Snacking is typically me |  |  |  |  |  |  |  |
| Snacking is something I have always done |  |  |  |  |  |  |  |

| Self-efficacy towards dog | Totally disagree |  |  | Neutral |  |  | Totally agree |
| --- | --- | --- | --- | --- | --- | --- | --- |
| I have a clear idea how to feed my dog less |  |  |  |  |  |  |  |
| I am capable of feeding my dog less |  |  |  |  |  |  |  |

| Support | Totally disagree |  |  | Neutral |  |  | Totally agree |
| --- | --- | --- | --- | --- | --- | --- | --- |
| Watching my dog’s weight motivates me to watch my own weight |  |  |  |  |  |  |  |

What do you feed your dog? (In percentage per day)

______ Dry food

______ Raw food

______ Sausages

______ Canned food

______ Table scraps

______ Home cooked

______ Other, please specify:

How often does your dog get a treat?

- More than 3 times per day
- Once per day
- Once per week
- Irregular
- Never

How do you feed your dog?

- Once a day, a fixed amount
- Twice a day, a fixed amount
- Three or more times a day, a fixed amount
- Ad libitum

Does the dog get leash walks?

- Yes, daily less than 1 hour
- Yes, daily for 1 to 3 hours
- Yes, daily more than 3 hours
- No, the dog walks on the premises (in the garden/surroundings)
- No

Appendix B Questionnaires at study end point

Human-centered trial visit 2

**Questions about the owner themselves**

| Quote | Totally disagree |  |  | Neutral |  |  | Totally agree |
| --- | --- | --- | --- | --- | --- | --- | --- |
| I know a lot about healthy diets |  |  |  |  |  |  |  |
| I know a lot about healthy exercise |  |  |  |  |  |  |  |
| I can resist temptations |  |  |  |  |  |  |  |
| My eating habits are healthy |  |  |  |  |  |  |  |
| I am lazy |  |  |  |  |  |  |  |
| I’m good at achieving long term goals |  |  |  |  |  |  |  |
| I like to snack |  |  |  |  |  |  |  |
| I sometimes don’t realize I’m snacking |  |  |  |  |  |  |  |

How often do you snack?

- More than 3 times per day
- Once to 3 times per day
- Once a day
- Irregularly
- Never

Do you walk/cycle/do sports (alone or with your dog)?

- Yes, daily for more than 3 hours
- Yes, daily for 1 to 3 hours
- Yes, daily for 1 hour
- Hardly
- Never

**Questions about the dog**

| Quote | Totally disagree |  |  | Neutral |  |  | Totally agree |
| --- | --- | --- | --- | --- | --- | --- | --- |
| My dog is healthy |  |  |  |  |  |  |  |
| My dog gets treats |  |  |  |  |  |  |  |

**Questions about the human-animal bound**

| Quote | Totally disagree |  |  | Neutral |  |  | Totally agree |
| --- | --- | --- | --- | --- | --- | --- | --- |
| My dog is my best friend |  |  |  |  |  |  |  |
| I talk to my dog |  |  |  |  |  |  |  |
| I think I lost more weight because of my dog |  |  |  |  |  |  |  |
| My dog supports me when I struggle |  |  |  |  |  |  |  |
| My dog experiences support from me |  |  |  |  |  |  |  |
| I am responsible for my dog’s weight |  |  |  |  |  |  |  |
| My dog snacks when I do |  |  |  |  |  |  |  |
| Sometimes I don’t realize I give my dog a treat |  |  |  |  |  |  |  |
| My dog understands me |  |  |  |  |  |  |  |

| Quote | Totally disagree |  |  | Neutral |  |  | Totally agree |
| --- | --- | --- | --- | --- | --- | --- | --- |
| Watching my own weight motivated me to watch my dog’s weight |  |  |  |  |  |  |  |
| Watching my dog’s weight motivated me to watch my own weight |  |  |  |  |  |  |  |

What do you feed your dog? (In percentages per day)

______ Dry food

______ Raw food

______ Sausages

______ Canned food

______ Table scraps

______ Home cooked

______ Other, please specify:

How often does your dog get a treat?

- More than 3 times per day
- Once per day
- Once per week
- Irregular
- Never

How do you feed your dog?

- Once a day, a fixed amount
- Twice a day, a fixed amount
- Three or more times a day, a fixed amount
- Ad libitum

Does the dog get leash walks?

- Yes, daily less than 1 hour
- Yes, daily for 1 to 3 hours
- Yes, daily more than 3 hours
- No, the dog walks on the premises (in the garden/surroundings)
- No

**Questions about the instructions for the owner**

| Quote | Totally disagree |  |  | Neutral |  |  | Totally agree |
| --- | --- | --- | --- | --- | --- | --- | --- |
| I followed the dietary instructions |  |  |  |  |  |  |  |
| It was difficult for me to eat healthier (or less unhealthy) food |  |  |  |  |  |  |  |
| I have eaten healthier (or less unhealthy) food this period |  |  |  |  |  |  |  |
| I followed the exercise instructions |  |  |  |  |  |  |  |
| I struggled to meet the exercise instructions |  |  |  |  |  |  |  |
| I have exercised more this period |  |  |  |  |  |  |  |

**Questions about the instructions for the dog (only for owner+dog group)**

| Not applicable | Quote | Totally disagree |  |  | Neutral |  |  | Totally agree |
| --- | --- | --- | --- | --- | --- | --- | --- | --- |
|  | I followed the dietary instructions |  |  |  |  |  |  |  |
|  | It was difficult for me to follow the dietary instructions |  |  |  |  |  |  |  |
|  | My dog has eaten healthier this period |  |  |  |  |  |  |  |
|  | I followed the exercise instructions |  |  |  |  |  |  |  |
|  | It was difficult to follow the exercise instructions |  |  |  |  |  |  |  |
|  | My dog exercised more this period |  |  |  |  |  |  |  |

**Questions about support**

| Not applicable | Quote | Totally disagree |  |  | Neutral |  |  | Totally agree |
| --- | --- | --- | --- | --- | --- | --- | --- | --- |
|  | I think I ate healthier because of my dog |  |  |  |  |  |  |  |
|  | I think I exercised more because of my dog |  |  |  |  |  |  |  |
|  | My dog ate healthier because of me |  |  |  |  |  |  |  |
|  | My dog exercised more because of me |  |  |  |  |  |  |  |
|  | Losing weight together with my dog was easier than alone |  |  |  |  |  |  |  |
|  | My dog supported me during weight loss |  |  |  |  |  |  |  |
|  | My dog experienced support from me during weight loss |  |  |  |  |  |  |  |
|  | My dog made it more difficult to eat healthy |  |  |  |  |  |  |  |
|  | My dog made it difficult for me to exercise more |  |  |  |  |  |  |  |

**Questions about the weight loss trial**

| Not applicable | Quote | Totally disagree |  |  | Neutral |  |  | Totally agree |
| --- | --- | --- | --- | --- | --- | --- | --- | --- |
|  | The trial positively affected my health |  |  |  |  |  |  |  |
|  | The trial motivated me to eat healthier |  |  |  |  |  |  |  |
|  | The trial motivated me to exercise more |  |  |  |  |  |  |  |

| Not applicable | Quote | Totally disagree |  |  | Neutral |  |  | Totally agree |
| --- | --- | --- | --- | --- | --- | --- | --- | --- |
|  | The trial positively affected my dog’s health |  |  |  |  |  |  |  |
|  | The trial motivated me to feed my dog healthier food |  |  |  |  |  |  |  |
|  | The trial motivated me to exercise my dog more |  |  |  |  |  |  |  |

**Questions about other sources of support**

| Not applicable | Quote | Totally disagree |  |  | Neutral |  |  | Totally agree |
| --- | --- | --- | --- | --- | --- | --- | --- | --- |
|  | My family and friends supported me during the trial |  |  |  |  |  |  |  |
|  | Participation in the research project supported me during the trial |  |  |  |  |  |  |  |
|  | I experienced equal or more support from my dog compared to family and friends |  |  |  |  |  |  |  |
|  | I experienced equal or more support from my dog compared to participation in the research project |  |  |  |  |  |  |  |

| **Concluding remarks:** |
| --- |

Dog-centered trial visit 2

| **Questions about human-animal bound** | Totally disagree | Disagree | Neutral | Agree | Strongly agree |
| --- | --- | --- | --- | --- | --- |
| My dog is more important to me than any person in the world |  |  |  |  |  |
| I share my feelings and concerns with my dog |  |  |  |  |  |
| Dogs should have the same rights as other family members |  |  |  |  |  |
| My dog is my best friend |  |  |  |  |  |
| My feelings toward people are influenced by their attitude towards my dog |  |  |  |  |  |
| I love my dog, because (s)he is more loyal to me than any person in the world |  |  |  |  |  |
| I love to share pictures of my dog |  |  |  |  |  |
| My dog is more than just a pet |  |  |  |  |  |
| I love my dog because (s)he never judges me |  |  |  |  |  |
| My dog knows when I’m feeling down |  |  |  |  |  |
| I talk a lot with other people about my dog |  |  |  |  |  |
| My dog understands me |  |  |  |  |  |
| Loving my dog keeps me healthy |  |  |  |  |  |
| Dogs earn the same amount of respect as people |  |  |  |  |  |
| Me and my dog are very close |  |  |  |  |  |
| I’ll do almost anything to care for my dog |  |  |  |  |  |
| I often play with my dog |  |  |  |  |  |
| My dog is my best companion |  |  |  |  |  |
| My dog makes me feel happy |  |  |  |  |  |
| My dog is part of my family |  |  |  |  |  |
| I am very much attached to my dog |  |  |  |  |  |
| Owning a dog contributes to my wellbeing |  |  |  |  |  |
| I consider my dog as a friend |  |  |  |  |  |

|  | Totally disagree |  |  | Neutral |  |  | Totally agree |
| --- | --- | --- | --- | --- | --- | --- | --- |
| My dog sleeps in my bed |  |  |  |  |  |  |  |
| I like to talk to my dog |  |  |  |  |  |  |  |
| I have conversations with my dog |  |  |  |  |  |  |  |
| I spend a lot of time preparing my dog’s meal |  |  |  |  |  |  |  |
| I am willing to spend a lot of money on my dog to make him look good |  |  |  |  |  |  |  |

I see my dog as a:

- Animal
- Friend
- Family member
- Child
- Baby
- Other, please specify: ____________________

| Responsibility | Totally disagree |  |  | Neutral |  |  | Totally agree |
| --- | --- | --- | --- | --- | --- | --- | --- |
| I am disappointed if I am unable to let my dog lose weight |  |  |  |  |  |  |  |
| It is important for me that my dog loses weight |  |  |  |  |  |  |  |
| It bothers me if I will not succeed in letting my dog lose weight |  |  |  |  |  |  |  |

| Responsibility | Totally disagree |  |  | Neutral |  |  | Totally agree |
| --- | --- | --- | --- | --- | --- | --- | --- |
| To which extend do you feel responsible for your dog’s weight? |  |  |  |  |  |  |  |
| To which extend do you regard your behavior responsible for your dog’s weight? |  |  |  |  |  |  |  |
| To which extend do you feel responsible for your own weight? |  |  |  |  |  |  |  |
| To which extend do you regard your behavior responsible for your own weight? |  |  |  |  |  |  |  |

| Self-efficacy towards themselves | Totally disagree |  | | Neutral | |  | | Totally agree |
| --- | --- | --- | --- | --- | --- | --- | --- | --- |
| I can resist temptations |  |  | |  | |  | |  |
| I find it difficult to stop bad habits |  |  | |  | |  | |  |
| I am lazy |  |  | |  | |  | |  |
| I say inappropriate things |  |  | |  | |  | |  |
| I sometimes do things that are bad to me for pleasure |  |  | |  | |  | |  |
| I refuse to do things that are bad to me |  |  | |  | |  | |  |
| I wish I had more discipline |  |  |  | |  | |  | |
| People say I am very disciplined |  |  |  | |  | |  | |
| Pleasures distract me to finish my (home)work |  |  |  | |  | |  | |
| I have difficulties focussing |  |  |  | |  | |  | |
| I am able to achieve long term goals |  |  |  | |  | |  | |
| Sometimes I cannot withhold myself from doing something which is bad to me |  |  |  | |  | |  | |
| I often do things without thinking about possible alternatives |  |  |  | |  | |  | |

| Habit-strength towards dog | Totally disagree |  |  | Neutral |  |  | Totally agree |
| --- | --- | --- | --- | --- | --- | --- | --- |
| I often give my dog treats |  |  |  |  |  |  |  |
| I give my dogs treats without being aware of it |  |  |  |  |  |  |  |
| I give my dog treats without thinking |  |  |  |  |  |  |  |
| I give my dog treats without needing a reminder |  |  |  |  |  |  |  |
| It feels strange if I don’t give my dog treats |  |  |  |  |  |  |  |
| It will take effort not to give my dog treats |  |  |  |  |  |  |  |
| Giving my dog treats is part of my daily/weekly routine |  |  |  |  |  |  |  |
| Giving my dog treats is typically me |  |  |  |  |  |  |  |
| Giving my dog treats is something I do for a very long time |  |  |  |  |  |  |  |

| Habit-strength towards themselves | Totally disagree |  |  | Neutral |  |  | Totally agree |
| --- | --- | --- | --- | --- | --- | --- | --- |
| Snacking is something I often do |  |  |  |  |  |  |  |
| Snacking is something I automatically do |  |  |  |  |  |  |  |
| Snacking is something I do without being aware of it |  |  |  |  |  |  |  |
| I feel awkward if I don’t take a snack |  |  |  |  |  |  |  |
| Snacking is something I do without thinking |  |  |  |  |  |  |  |
| Stop snacking will be an effort for me |  |  |  |  |  |  |  |
| Snacking belongs to my daily/weekly routine |  |  |  |  |  |  |  |
| I start snacking before thinking about it |  |  |  |  |  |  |  |
| Stop snacking will be difficult for me |  |  |  |  |  |  |  |
| I start snacking before being aware of it |  |  |  |  |  |  |  |
| Snacking is typically me |  |  |  |  |  |  |  |
| Snacking is something I do for a very long time |  |  |  |  |  |  |  |

| Self-efficacy towards dog | Totally disagree |  |  | Neutral |  |  | Totally agree |
| --- | --- | --- | --- | --- | --- | --- | --- |
| I have a clear idea how to feed my dog less |  |  |  |  |  |  |  |
| I am capable of feeding my dog less |  |  |  |  |  |  |  |

| Support | Totally disagree |  |  | Neutral |  |  | Totally agree |
| --- | --- | --- | --- | --- | --- | --- | --- |
| Watching my dog’s weight motivates me to watch my own weight |  |  |  |  |  |  |  |

What do you feed your dog? (In percentage per day)

______ Dry food

______ Raw food

______ Sausages

______ Canned food

______ Table scraps

______ Home cooked

______ Other, please specify:

How often does your dog get a treat?

- More than 3 times per day
- Once per day
- Once per week
- Irregular
- Never

How do you feed your dog?

- Once a day, a fixed amount
- Twice a day, a fixed amount
- Three or more times a day, a fixed amount
- Ad libitum

Does the dog get leash walks?

- Yes, daily less than 1 hour
- Yes, daily 1 to 3 hours
- Yes, daily more than 3 hours
- No, the dog walks on the premises (in the garden/surroundings)
- No

Dog only group

| Questions about the trial | Totally agree |  |  | Neutral |  |  | Totally disagree |
| --- | --- | --- | --- | --- | --- | --- | --- |
| I followed the health guidelines for my dog |  |  |  |  |  |  |  |
| It was difficult for me to keep the health guidelines for my dog |  |  |  |  |  |  |  |
| Keeping the health guidelines for my dog was an effort |  |  |  |  |  |  |  |
| The trial had positive effects on my dog’s health |  |  |  |  |  |  |  |
| My dog has become healthier |  |  |  |  |  |  |  |
| The trial had positive effects on my own eating/exercise habits |  |  |  |  |  |  |  |
| I am healthier now |  |  |  |  |  |  |  |
| The trial motivated me to lose weight myself |  |  |  |  |  |  |  |

Dog+owner group

| Questions about the trial | Totally disagree |  |  | Neutral |  |  | Totally agree |
| --- | --- | --- | --- | --- | --- | --- | --- |
| I followed the health guidelines for my dog |  |  |  |  |  |  |  |
| It was difficult for me to follow the health guidelines for my dog |  |  |  |  |  |  |  |
| Following the health guidelines for my dog was an effort |  |  |  |  |  |  |  |
| The trial had positive effects on my dog’s health |  |  |  |  |  |  |  |
| My dog has become healthier |  |  |  |  |  |  |  |
| The trial had positive effects on my own eating/exercise habits |  |  |  |  |  |  |  |
| I am healthier now |  |  |  |  |  |  |  |
| The trial motivated me to lose weight myself |  |  |  |  |  |  |  |
| I followed the health guidelines for myself |  |  |  |  |  |  |  |
| It was difficult for me to follow the health guidelines |  |  |  |  |  |  |  |
| Following the health guidelines was an effort |  |  |  |  |  |  |  |
| Losing weight myself is easier compared to letting my dog lose weight |  |  |  |  |  |  |  |
